# Conflict resolution tactics between mothers and children in a capital city in southeastern Brazil: prevalence and correlations

**DOI:** 10.1590/1980-220X-REEUSP-2025-0351en

**Published:** 2026-04-03

**Authors:** Laura Fontes Silva, Franciéle Marabotti Costa Leite

**Affiliations:** 1 Universidade Federal do Espírito Santo, Programa de Pós-Graduação em Saúde Coletiva, Vitória, ES, Brazil.

**Keywords:** Family Conflict, Family Relations, Child Abuse, Sexual

## Abstract

**Objective:**

To identify the prevalence of conflict resolution tactics between mothers and children and their correlations.

**Method:**

This cross-sectional study included 418 women from Vitória, Espírito Santo, Brazil. The outcome was the conflict resolution tactics used by mothers with children under 19 years of age, measured by the Parent-Child Conflict Tactics Scales, categorized into non-violent discipline, psychological aggression, corporal punishment, and maltreatment. Analyses were performed using STATA and R, and presented as absolute and relative frequencies, mean, standard deviation, 95% confidence interval, Pearson’s chi-square test (χ^2^) or Fisher’s exact test, and correlation test.

**Results:**

There was a higher prevalence of the use of non-violent discipline (P: 88.8%; 95%CI: 85.7–91.8). Approximately 82% (95%CI: 78.1–85.5) used psychological aggression, 35.9% used corporal punishment (95%CI: 31.3–40.5), and 23.4% (95%CI: 19.4–27.5) used some disciplinary method that constitutes mistreatment.

**Conclusion:**

Although non-violent discipline is widely adopted by mothers, there is a significant presence of psychological aggression and physical assault, as well as practices of mistreatment.

## INTRODUCTION

Although parenting has undergone changes in recent times, the primary responsibility for raising children still falls predominantly on mothers^(1)^. Motherhood as we see it today is the result of changes in the family context that assign women the role of not only nurturing, but also caring for and guiding their children, often making them the primary caregiver^(2)^. Therefore, it is a complex concept that is not limited to a specific dimension, but is characterized as a multidimensional adaptation in a woman’s life that involves redefining body image and identity, attachment formation, well-being, changing priorities in relationships, and confidence in performing the role of mother, which allows women to integrate and balance themselves both as an individual and as a mother^(3)^.

High demands, a child’s behavior, and the adjustment period to the new role make the beginning of the parental journey challenging for parents^(4)^. The understanding that the way primary caregivers deal with their own issues in the context of childcare influences how children develop their emotional and social skills^(5)^, culminating in new health productions that guide parents on more conscious parenting practices based on mutual respect^(6)^.

It is well known that, in challenging contexts such as conflict, emotional intelligence becomes a central issue^(7)^. Conflicts are inherent in human relationships^(8)^, and the family environment often constitutes a space conducive to the emergence of disagreements, due to the inevitable daily divergences and tensions among its members^(9)^. Recurring conflicts between parents and children are characterized by frequent and irresolvable arguments between parents and children on the same topics^(10)^.

The reasons why mothers experience conflicts related to their children change throughout children’s development, as shown by a study with young people aged 15 to 24. This study identified that the main parental conflicts are between mothers and children, with the primary motivation being household chores, followed by money, and internet use. Other motivations such as friendship/dating, drugs, and studies are also presented in the research^(11)^. It is a fact that frequent conflicts can generate toxic stress, harming emotional and cognitive development and contributing to mental disorders in both parties involved^(12)^. These conflicts are often associated with intergenerational cycles of violence and neglect^(13)^.

In this context, the Statute of Children and Adolescents and Law 13,010/2014 are fundamental, as they guarantee the right to education and care without the use of physical punishment or cruel and degrading treatment for all children and adolescents, recognizing domestic violence as a violation of rights, not as a legitimate educational practice^(14,15)^. It is worth mentioning that, in 2014, the “Spanking Law” was enacted in Brazil, which criminalized any physical punishment with punitive or disciplinary intent, as well as cruel conduct that degrades, intimidates, or humiliates victims^(15)^.

However, despite the State’s efforts to implement measures to protect children and adolescents in situations of violence and neglect, the phenomenon has only increased over the years. According to the Brazilian Yearbook of Public Security, in the first half of 2021, Brazil had already registered more than 50,000 reports of violence against children and adolescents nationwide through the “Dial 100” hotline, a channel for reporting human rights violations in the country. In 81% of reports, the violence occurred in victims’ homes, and the main perpetrators were mothers, followed by fathers^(16)^. In Espírito Santo, from 2011 to 2018, 3,127 cases of violence against children were reported, with perpetrators mostly being people with maternal or paternal ties to the victim^(17)^.

It is important to reflect that the use of physical punishment in children can be modified through specific interventions based on parental support programs that aim to teach alternatives to raising children without the use of violence^(18)^. Law 14,826/2024 provides guidance on positive parenting and the right to play as tools for preventing violence against children. Initiatives based on criteria of respect, communication, and limits are now being used in social assistance, education, culture, health, and public safety throughout the country (Bill 2,861/2023)^(19)^.

Given the above, understanding the tactics used by mothers in resolving domestic conflicts is fundamental to public health, as it directly impacts children’s well-being and family dynamics. Knowing these patterns can help prevent domestic violence and promote mental health, offering adequate support to mothers in stressful situations. In this regard, the present study seeks to identify the prevalence of conflict resolution tactics between mothers and children and their correlations.

## METHOD

### Study Design

This is a cross-sectional, descriptive, and analytical study derived from a population-based study conducted in the municipality of Vitória, Espírito Santo, Brazil.

### Context

The municipality of Vitória, capital of the state of Espírito Santo, according to the 2022 census by the Brazilian Institute of Geography and Statistics (In Portuguese, *Instituto Brasileiro de Geografia e Estatística* - IBGE), has an area of 97,123 km^2^ and a resident population of 322,869 people; among them, 54% are women, indicating a total of approximately 174,348 female residents, distributed across 80 neighborhoods with a population density of 3,324,33 inhabitants/km^2(20)^.

The population-based study took place in 2022, from January to May, with the pilot study in 2021. Data collection was carried out in homes by teams composed of female interviewers and supervisors. Interviews were conducted with women using a structured questionnaire as a guide, applied in person in the woman’s residence, ensuring privacy. The collected data were processed on tablets and managed using the Research Electronic Data Capture tool.

### Participants

The target population of the study included women aged 18 or older who have or have had an intimate partner in the 24 months prior to the interview and who have children up to 19 years of age. An intimate partner was defined as a current or former partner, provided there was sexual involvement, regardless of whether they were formally married. As an exclusion criterion, women with cognitive limitations resulting from intellectual or sensory deficits that prevented them from responding to the data collection instrument were considered.

### Variables

The study outcome is the conflict between mothers and children, which was identified through the use of the Parent-Child Conflict Tactics Scales (CTSPC). This scale originally consists of 22 items that investigate reactions to conflict based on parental behavior towards children. The scale is validated for use in Brazil. Three dimensions are assessed: non-violent discipline (NVD) – (four items) – a tactic referring to correction methods based on explanations, dialogue, and withdrawal of privileges, without the use of physical force; psychological aggression – (five items) – consisting of verbal and symbolic acts that cause fear, humiliation, or emotional pain in the child, such as yelling, threatening, and insulting; physical assault – (13 items) – which includes the use of bodily force, ranging from mild punishments, such as spanking, to severe aggression, which constitutes abuse. The latter was subdivided according to severity into corporal punishment (six items), physical abuse (three items), and severe physical assault (four items)^(21)^.

For the present study, the measurement of each domain was done in a dichotomized manner (presence/absence of the conduct), with “yes” or “no” response options. Two questions related to severe physical abuse were excluded. Thus, in this research, the scale includes 20 items. The exclusion of the two questions related to severe physical abuse occurred after the pilot test, since the interviewers found that the questions related to the use of firearms and the use of hot liquids to injure their children generated discomfort and distrust in participating in the study. Therefore, they were excluded from the questionnaire.

Concerning independent variables, the following characteristics were assessed: age (18–29; 30–39; 40–49; 50–59; 60 or more); education in years of study (0–8; 9–11; 12 or more); family income (1^st^ tercile – poorest; 2^nd^ tercile and 3^rd^ tercile – richest); number of residents in the household (living alone; two; three; four; five or more people); number of pregnancies (one; two; three; four or more), number of children (one; two; three; four or more); history of abortion.

### Study Size

For the study’s sampling, the 2010 IBGE census was used to select census tracts corresponding to the cadastral unit by continuous area within a region, resulting in 108,515 households chosen in the region. These households were divided into one hundred, allowing for the random selection of the initial sector, and the subsequent 99 sectors were selected based on a systematic selection process. This resulted in a total of 1,086 women to be selected per household. It should be noted that, in the selection process, if there was more than one eligible woman in the residence, a numerical list was drawn for the random selection of one of them. This entire process was carried out using the R statistical software, taking into account the socioeconomic level classification. For this study, the sample consisted of mothers of children up to 19 years old residing in the municipality of Vitória, Espírito Santo, totaling 418 women.

### Statistical Methods

For descriptive analysis, absolute and relative frequencies, mean, standard deviation, and 95% Confidence Interval (95%CI) were used. In bivariate analysis, Pearson’s chi-square test (χ^2^) and Fisher’s exact test were applied to examine the relationship among variables. To test the correlation among the items that make up conflict tactics, Phi (φ) coefficients were calculated due to the nature of the variables. Data analysis was conducted using Stata software version 17.0, and the correlation matrix was performed using R software version 4.3.1, with an adjustment for p-value <0.05.

The research was approved by the *Universidade Federal do Espírito Santo* Research Ethics Committee, under Opinion 4.668206.

## RESULTS

The women participating in this study had a mean age of 41 years and a mean of 12 years of completed education. The mean family income was R$ 4,447.34, with a median of R$ 2,400, indicating an asymmetrical distribution of the variable. As for motherhood, the mean number of pregnancies was 2.45, and the mean number of children was 2.16. The mean number of people residing in the household was 3.63. As for reproductive history, the mean number of reported abortions was 1.32 (Table 1).

**Table 1:** Characterization of the sample of mothers participating in the study in Vitória, ES, Brazil, 2022 (N = 418).

	Mean	Standard deviation	Median	Interval
Age	41.03	± 11.81	40.0	33–47
Education level	12.03	± 3.89	11.0	10–15
Family income (R$)	4447.34	± 5029.67	2400	1250–6000
Number of pregnancies	2.45	± 1.68	2.0	1–3
Number of children	2.16	± 1.60	2.0	1–3
Number of people living in the house	3.63	± 1.36	3.05	3–4
Abortion	1.32	± 0.68	1.0	1–1

In relation to the NVD outcome, the majority (88.8%) of mothers explained to their children why their actions were wrong (95%CI: 85.7–91.8); 76.8% (95%CI: 72.7–80.1) put their children in time-out, such as in their room or another location; 60.8% (95%CI: 56.1–65.4) gave them another activity to replace the wrong action; and 68.9% (95%CI: 64.5–73.3) withdrew privileges or restricted outings. Concerning psychological aggression, 81.8% of mothers resorted to yelling or screaming (95%CI: 78.1–85.5). Moreover, 14.8% cursed or swore at their children (95%CI: 11.4–18.2). 9.8% (95%CI: 6.9–12.6) threatened to kick the child out of the house; 61.7% (95%CI: 57.1–66.4) threatened to slap the child but did not; and 25.4% (95%CI: 21.2–29.5) called their children donkeys, stupid, or similar (Table 2).

**Table 2:** Conflict resolution tactics regarding non-violent discipline and psychological aggression used by mothers residing in Vitória, ES, Brazil, 2022 (N = 418).

Non-violent discipline	n	%	95%CI
Did you explain why what they were doing was wrong?	371	88.8	85.7–91.8
Did you punish them by making them stay in their room or somewhere else?	321	76.8	72.7–80.1
Did you give them something else to do instead of what they were doing wrong?	254	60.8	56.1–65.4
Did you take away their privileges or leave them confined to the house?	288	68.9	64.5–73.3
**Psychological aggression**	**n**	**%**	**95%CI**
Did you speak loudly, yell, or shout at your child?	342	81.8	78.1–85.5
Did you curse or swear at them, i.e., did you put a curse on them?	62	14.8	11.4–18.2
Did you ever say you were going to kick them out of the house or shoo them out of the house?	41	9.8	6.9–12.6
You threatened to slap them, but did not?	258	61.7	57.1–66.4

Legend: 95%CI – 95% Confidence Interval; n – number; % – percentage.

In relation to corporal punishment, 35.9% (95%CI: 31.3–40.5) shook the child; 56.2% (95%CI: 51.5–61.0) hit with objects such as a belt or slipper; 70.8% (95%CI: 66.5–75.2) spanked the buttocks; 49.5% (95%CI: 44.7–54.3) slapped the hands, arms, or legs; and 30.9% pinched (95%CI: 26.4–35.3). As for physical abuse, 6.7% (95%CI: 4.3–9.1) hit with a closed fist or kicked the child; 23.4% (95%CI: 19.4–27.5) used objects to hit other parts of the body besides the buttocks; 2.2% (95%CI: 0.8–3.5) threw the child down or grabbed the child by the neck and shook them; and about 3% reported hitting the child continuously and intensely (95%CI: 1.3–4.5) (Table 3).

**Table 3:** Conflict resolution tactics related to physical assault used by mothers residing in Vitória, ES, Brazil, 2022 (N = 418).

	Physical assault	n	%	95%CI
Corporal punishment	Did you shake them?	150	35.9	31.3–40.5
	Did you hit their bottom with something, like a belt, slipper, hairbrush, stick, or other hard object?	235	56.2	51.5–61.0
	Did you spank someone’s bottom?	296	70.8	66.5–75.2
	Did you slap their hand, arm, or leg?	207	49.5	44.7–54.3
	Did you pinch them?	129	30.9	26.4–35.3
Physical abuse	Did you hit them with a closed fist or kick them hard?	28	6.7	4.3–9.1
	Did you hit any part of their body other than their buttocks with something like a belt, flip-flop, hairbrush, stick, or other hard object?	98	23.4	19.4–27.5
	Did you throw them down?	9	2.2	0.8–3.5
	Did you grab them by the neck and shake them?	9	2.2	0.8–3.5
	Did you hit them a lot, meaning did you hit them nonstop, as much as you could?	12	2.9	1.3–4.5

Legend: 95%CI – 95% Confidence Interval; n – number; % – percentage.

**Figure 1:**
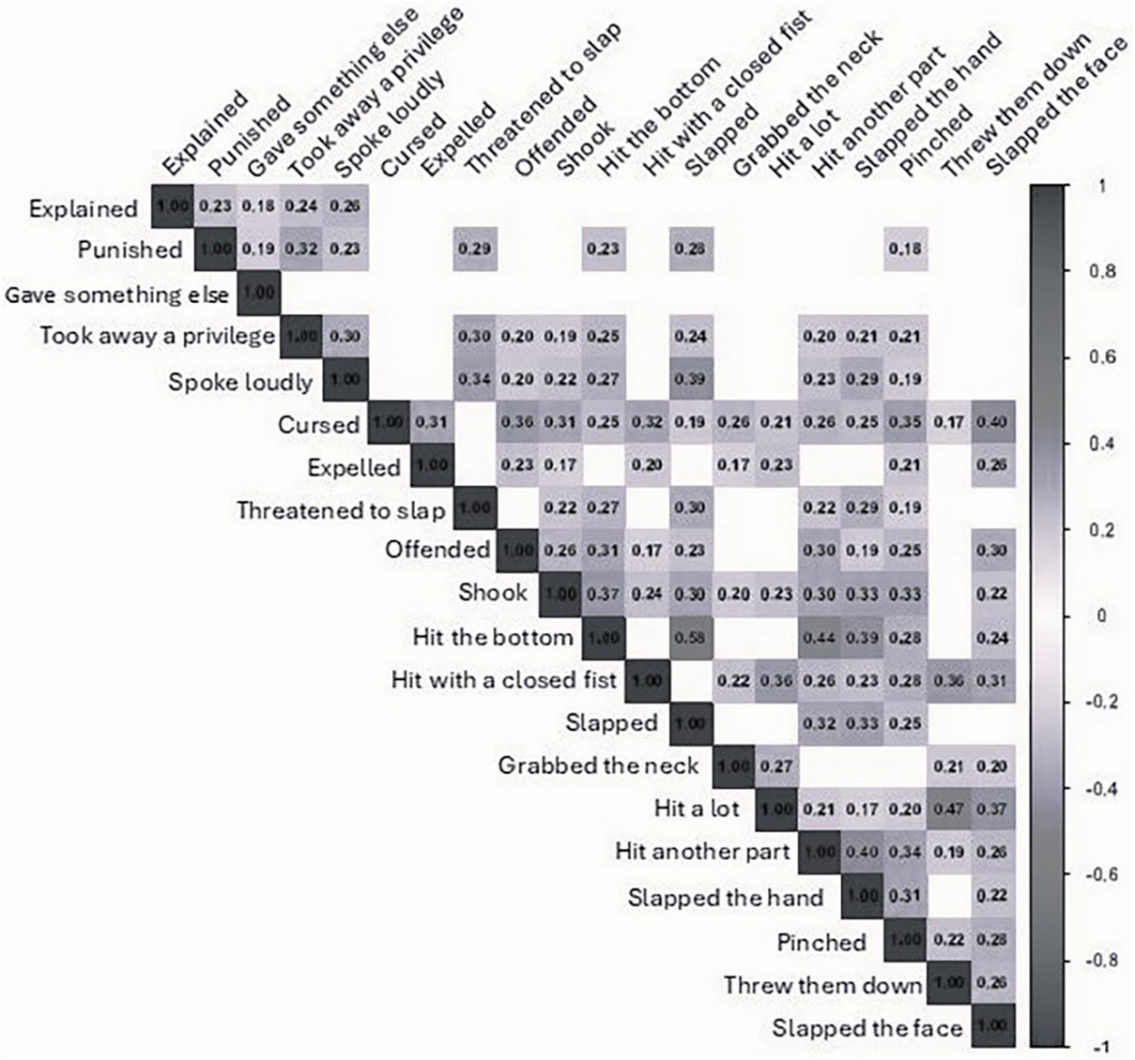
Correlation matrix between the conflict tactics of mothers residing in the municipality of Vitória, ES, Brazil, 2022 (N = 418).

Figure 1 presents the correlation among conflict tactics. The analysis demonstrated that some variables show a positive correlation, such as “swearing” with “slap in the face” (0.40), “hit the butt” with “slap” (0.58), “hit the butt” with “hit other parts of the body different from the butt” (0.44), “hit a lot” with “threw them down” (0.47) and “hit other parts of the body different from the butt” with “slap on the hand” (0.40).

## DISCUSSION

The findings of this study show that NVD is the practice predominantly adopted by the mothers in the study. The results of the analysis support those of a similar study in southern Brazil with parents of children aged 4 to 6 years, where the practice of NVD by mothers was also the most prevalent, being used by 60% of mothers^(22)^.

Other studies, such as those analyzing conflict resolution tactics in specific locations across various countries, have found a positive response rate to NVD of up to 98%. The Philippines leads in this practice, with 97% acceptance, followed by Chile with 96%, and then Brazil, also with 96%, based on data from the city of São Paulo. However, despite prevailing in all countries analyzed, positive practices are accompanied by violent conflict resolution tactics, highlighting the harsh treatment of children as an epidemic problem in all the communities studied^(23)^.

Furthermore, in the present study, it was found that the majority of participants (81.8%) resorted to at least one form of psychological aggression. Regarding psychological abuse, the results corroborate research in emergency services in the capital of the state of Rio de Janeiro, where the prevalence of psychological aggression was 94.8%. The authors draw attention to the fact that, despite the high prevalence of psychological aggression, the reporting of this type of violence seems to be almost non-existent in the state of Rio de Janeiro^(24)^.

The results identify a high prevalence of physical assault, with approximately 71% reporting at least one act of corporal punishment. A similar study conducted in the Portuguese population shows a positive response rate of 87.7% for at least one act of corporal punishment, lower only than the practice of psychological aggression in the seven-year-old population^(25)^. These data show that despite being criminalized in the country since 2007, corporal punishment is, according to parents, related to the act of educating^(26)^.

Studies indicate that legal prohibition alone has limited effectiveness and must be accompanied by educational initiatives, as well as the promotion of positive parenting, for effective change^(27)^. Furthermore, other studies have already proven that corporal punishment is an inefficient disciplinary method that tends to produce immediate obedience, but does not foster emotional self-regulation or awareness of one’s actions^(28)^. This fact reinforces that corporal punishment, besides not being a cohesive method of education, represents a violation of the rights of children and adolescents, presenting itself as a public health problem with lasting and severe consequences, which demands integrated policies of prevention, psychosocial support and strengthening of parental skills^(29)^.

The same is observed in a study in Diamantina, a city in Minas Gerais located in southeastern Brazil, which found that violence is frequently accompanied by justifications that make it seem natural in everyday life, being interpreted as a common and banalized practice in our society. Violence is seen as a legitimate and acceptable instrument of discipline, which contributes to its perpetuation and hinders the recognition of its harmful impacts^(30)^.

Child abuse was present in at least 23.4% of participants’ responses. A study with a population from the Southeast region of Brazil indicates that 50.3% of mothers practiced at least one type of child abuse as a conflict resolution tactic^(31)^. Another study in China found that the response rate to mistreatment practices was 72.5%, suggesting that tolerance for corporal punishment of children in the East is culturally higher compared to Western society^(32)^.

It is worth highlighting that despite the high prevalence of NVD use, its practice has been observed concurrently with other conflict resolution tactics, as indicated by the authors of the CTSPC during its validation, reinforcing that parents tend to use more than one tactic to control their child’s behavior. They also note that corporal punishment is a popular and socially accepted practice^(8)^. This finding is verified in the present research, which indicates a positive correlation between practices of physical and psychological aggression.

Most families combine practices of physical maltreatment with some form of physical and emotional abuse. Any arrangement of practices involving maltreatment deserves attention, regardless of the frequency. This is because child maltreatment has the greatest impact on children, affecting their enjoyment of life and influencing their future and outlook^(33,34)^. Healthcare professionals must be aware of indicators of abuse and their specific characteristics in order to develop effective strategies and policies to combat this type of violence.

The use of any aggressive, coercive practice is linked to various health problems throughout life, including mental health issues, poorer parent-child relationships, substance use, impaired socio-emotional development, negative academic performance, increased externalizing behaviors, and more^(35)^.

Since this is a cross-sectional study, there is a possibility of recall bias, since the data were obtained through self-reporting, depending on participants’ recollection of past events, which may underestimate the findings. The study is also subject to information bias due to the provision of socially desirable responses. Furthermore, it is worth mentioning that the data refers to a capital city in Brazil, and the study is not able to apply this data to a national level. However, even with these limitations, the findings are highly relevant and reveal the magnitude of conflict tactics between mothers and children and the importance of further studies on this topic.

## CONCLUSION

Although the results reveal that NVD is widely adopted, there is a significant presence of psychological aggression and physical assault in the conflict resolution tactics between mothers and children, particularly corporal punishment and mistreatment. This finding is concerning because it weakens emotional bonds and can have lasting effects on the child’s mental health and behavior, as well as impacting how children cope with conflict situations, as they never know what to expect from caregivers as a solution. Furthermore, experiencing violence can impair the ability to establish healthy and secure relationships, perpetuating cycles of suffering and social exclusion.

Finally, this study not only points to the need for further research, but also highlights the importance of public awareness campaigns aimed at disseminating legislation that prohibits corporal punishment. These campaigns, when combined with educational programs that guide parents on non-violent and respectful disciplinary practices, act as catalysts for change in parents’ beliefs and behaviors regarding the use of coercive disciplinary strategies.

## Data Availability

The entire dataset supporting the results of this study is available upon request to the corresponding author.
