# Supplementary material for: Conflict resolution tactics between mothers and children in a capital city in southeastern Brazil: prevalence and correlations
Source: Rev Esc Enferm USP. 2026 Apr 3;60:e20250351. doi: 10.1590/1980-220X-REEUSP-2025-0351en (PMC13074426; doi:10.1590/1980-220X-REEUSP-2025-0351en)
Supplement: Translation

# Translation: Táticas de resolução de conflitos entre mães-filhos em capital do Sudeste: prevalências e correlações

**DOI:** 10.1590/1980-220X-REEUSP-2025-0351pt

**Authors:** Laura Fontes Silva, Franciéle Marabotti Costa Leite

**Affiliations:** 1 Universidade Federal do Espírito Santo, Programa de Pós-Graduação em Saúde Coletiva, Vitória, ES, Brasil.

**Keywords:** Conflito Familiar, Relações Familiares, Abuso Sexual na Infância

## Abstract

**Objetivo:**

Identificar as prevalências das táticas de resolução de conflitos entre mães-filhos e suas correlações.

**Método:**

Estudo transversal, com 418 mulheres de Vitória, Espírito Santo. O desfecho foi a tática de resolução de conflitos utilizada pelas mães com filhos menores de 19 anos, mensurado pelo *Parent-Child Conflict Tactics Scales*, categorizado em disciplina não violenta, agressão psicológica, punição corporal e maus-tratos. As análises foram realizadas no STATA e R, e apresentadas em frequência absoluta, relativa, média, desvio padrão, Intervalo de Confiança de 95%, teste qui-quadrado de Pearson (χ^2^) ou teste exato de Fisher, e teste de correlação.

**Resultados:**

Houve maior prevalência do uso de disciplina não violenta (P: 88,8%; IC95%: 85,7–91,8). Cerca de 82% (IC95%: 78,1–85,5) fizeram uso de agressão psicológica, 35,9%, de punição corporal (IC95%: 31,3–40,5), e 23,4% (IC95%: 19,4–27,5) usaram algum método disciplinar que configura maus-tratos.

**Conclusão:**

Embora a disciplina não violenta seja amplamente adotada pelas mães, há uma presença significativa de agressão psicológica e física, além de práticas de maus-tratos.

## INTRODUÇÃO

Embora a parentalidade tenha passado por mudanças na atualidade, a principal responsabilidade pela criação dos filhos ainda recai, predominantemente, sobre as mães^(1)^. A maternidade como vemos hoje é fruto de mudanças no contexto familiar que atribuem à mulher o papel de não só nutrir, mas também de zelar e orientar seus filhos, sendo, na maioria das vezes, a principal cuidadora^(2)^. Trata-se, portanto, de um conceito complexo que não se limita a uma dimensão específica, mas caracteriza-se como uma adaptação multidimensional na vida da mulher que envolve a redefinição da imagem corporal e identidade, a formação de apego, o bem-estar, a mudança de prioridades nos relacionamentos, e a confiança no desempenho do papel de mãe, o que possibilita que a mulher se integre e se equilibre tanto como indivíduo quanto como mãe^(3)^.

A alta demanda, o comportamento da criança e o período de adaptação ao novo papel tornam o início da jornada parental desafiadora aos pais^(4)^. O entendimento de que a forma como os principais cuidadores lidam com suas próprias questões diante do cuidado com os filhos influencia a forma como as crianças desenvolvem suas habilidades emocionais e sociais^(5)^, culminando em novas produções em saúde que orientam os sobre pais práticas parentais mais conscientes e pautadas no respeito mútuo^(6)^.

É notório que, em contextos desafiadores como o de conflitos, a habilidade emocional se torna uma questão central^(7)^. Os conflitos são inerentes às relações humanas^(8)^, e o ambiente familiar frequentemente constitui um espaço propício para o surgimento de desentendimentos, devido às divergências e tensões cotidianas inevitáveis entre seus integrantes^(9)^. Conflitos recorrentes entre pais e filhos são caracterizados por discussões frequentes e irresolvíveis entre pais e filhos sobre os mesmos tópicos^(10)^.

Os motivos pelos quais as mães vão tendo conflitos relacionados ao filho vão mudando no decorrer do desenvolvimento da criança, como constatado por estudo com jovens de 15 a 24 anos que identificou que os principais conflitos parentais são entre mães e filhos, sendo a principal motivação os afazeres domésticos, seguida por dinheiro e uso de internet. Outras motivações como amizade/namoro, drogas e estudos também são apresentadas na pesquisa^(11)^. É fato que conflitos frequentes podem gerar estresse tóxico, prejudicando o desenvolvimento emocional e cognitivo, contribuindo para transtornos mentais em ambos os envolvidos^(12)^. Esses conflitos frequentemente estão associados a ciclos intergeracionais de violência e negligência^(13)^.

Nesse contexto, o Estatuto da Criança e Adolescente e a Lei nº 13.010/2014 são fundamentais, pois asseguram o direito à educação e cuidados sem o uso de castigos físicos ou tratamentos cruéis e degradantes a todas as crianças e adolescentes, reconhecendo a violência doméstica como violação de direitos, não como prática educativa legítima^(14,15)^. Vale mencionar que, em 2014, foi sancionada no Brasil a Lei da Palmada, onde passou a ser crime qualquer castigo físico com intencionalidade punitiva ou disciplinar e, da mesma forma, as condutas cruéis que venham a degradar, intimidar ou humilhar as vítimas^(15)^.

Entretanto, apesar dos esforços do Estado em implementar medidas que assegurem a crianças e adolescentes em situação de violência e negligência, o fenômeno só tem aumentado com o passar dos anos. Segundo o Anuário Brasileiro de Segurança Pública, no primeiro semestre de 2021, o Brasil já havia registrado mais de 50.000 denúncias de violência contra crianças e adolescentes no território nacional através do “Disque 100”, canal de denúncia de violações dos direitos humanos no país. Em 81% das denúncias, as violências aconteceram na casa das vítimas, e os maiores agressores foram as mães, seguidas pelos pais^(16)^. No Espírito Santo, de 2011 a 2018, 3.127 casos de violência contra a criança foram notificados, sendo os agressores, em sua maioria, pessoas com vínculo materno e paterno com a vítima^(17)^.

É importante refletir que a aplicação de punições físicas em crianças pode ser modificada por meio de intervenções específicas pautadas em programas de apoio parental que visam ensinar alternativas à criação sem o uso de violência^(18)^. A Lei nº 14.826/2024 orienta sobre a parentalidade positiva e o direito de brincar como ferramentas de prevenção de violência contra crianças. As iniciativas com fundamentações apoiadas em critérios de respeito, comunicação e limites passam a ser utilizadas na assistência social, educação, cultura, saúde e segurança pública em todo o país (Projeto de Lei nº 2.861/2023)^(19)^.

Diante do exposto, a compreensão das táticas utilizadas por mães na resolução de conflitos domésticos é fundamental para a saúde pública, por impactar diretamente o bem-estar das crianças e a dinâmica familiar. Conhecer esses padrões pode auxiliar na prevenção de violência doméstica e na promoção de saúde mental, oferecendo suporte adequado a mães em situações de estresse. Nesse sentido, o presente estudo busca identificar as prevalências das táticas de resolução de conflitos entre mães-filhos e suas correlações.

## MÉTODO

### Desenho do Estudo

Trata-se de pesquisa de delineamento transversal, descritivo e analítico, oriunda de estudo de base populacional realizado no município de Vitória, Espírito Santo, Brasil.

### Contexto

O município de Vitória, capital do estado do Espírito Santo, segundo o censo do Instituto Brasileiro de Geografia e Estatística (IBGE) de 2022, tem uma área de 97,123 km^2^ e uma população residente de 322.869 pessoas; entre elas, 54% são mulheres, o que indica um total aproximado de 174.348 mulheres residentes, distribuídas em 80 bairros com uma densidade demográfica de 3.324,33 hab./km^2(20)^.

O estudo de base populacional aconteceu no ano de 2022, de janeiro a maio, sendo o estudo piloto em 2021. A coleta de dados foi realizada em domicílio por equipes compostas por entrevistadoras do sexo feminino e supervisores. Foram realizadas entrevistas com as mulheres, utilizando-se um questionário estruturado como roteiro, aplicado face-a-face na residência da mulher, garantindo a privacidade. Os dados coletados foram computados em *tablets* e gerenciados por auxílio da ferramenta eletrônica de captura de dados *Research Eletronic Data Capture*.

### Participantes

A população-alvo do estudo incluiu mulheres com 18 anos ou mais que tenham ou tenham tido um parceiro íntimo nos 24 meses anteriores à entrevista e que tenham filhos com até 19 anos de idade. O parceiro íntimo foi definido como o atual ou ex-parceiro, desde que houvesse envolvimento sexual, independentemente de estarem formalmente unidos. Como critério de exclusão, foram consideradas mulheres que apresentavam limitações cognitivas decorrentes de déficits intelectuais ou sensoriais, que as impediam de responder ao instrumento de coleta de dados.

### Variáveis

O desfecho do estudo são os conflitos entre mães e filhos, que foi identificado por meio da utilização da *Parent-Child Conflict Tactics Scales* (CTSPC), que se constitui originalmente por 22 itens que investigam as reações ao conflito a partir do comportamento parental dos pais em direção aos filhos. A escala é validada para uso no Brasil. Três dimensões são avaliadas: disciplina não violenta (DNV) - (quatro itens) tática referente a métodos de correção baseados em explicações, diálogo e retirada de privilégios, sem o uso de força física; agressão psicológica - (cinco itens) constituída por atos verbais e simbólicos que causam medo, humilhação ou dor emocional na criança, como gritar, ameaçar e insultar; violência física - (13 itens) que compreende o uso de força corporal, variando de punições leves, como palmadas, até agressões graves, que configuram maus-tratos. A última foi subdividida conforme a gravidade em punição corporal (seis itens), maus-tratos físicos (três itens) e maus-tratos físicos graves (quatro itens)^(21)^.

Para o presente estudo, a mensuração de cada domínio foi feita de forma dicotomizada (presença/ausência da conduta), com opções de respostas “sim” ou “não”. Foram excluídas duas questões relacionadas a maus-tratos físicos graves. Dessa forma, na presente pesquisa, a escala contempla 20 itens perguntados. A exclusão das duas questões relacionadas aos maus-tratos físicos graves aconteceu após o teste piloto, uma vez que foi constatado pelas entrevistadoras que as perguntas relacionadas ao uso de arma de fogo e uso de líquido quente para ferir seus filhos geravam desconforto e desconfiança na participação no estudo. Assim, foram excluídas do questionário.

Em relação às variáveis independentes, foram avaliadas as seguintes características: idade (18–29; 30–39; 40–49; 50–59; 60 ou mais); escolaridade em anos de estudo (0–8; 9–11; 12 ou mais); renda familiar (1º tercil – mais pobre; 2º tercil e 3º tercil – mais rico); número de moradores no domicílio (morando sozinho; duas; três; quatro; cinco ou mais pessoas); número de gestações (uma; duas; três; quatro ou mais), número de filhos (um; dois; três; quatro ou mais); histórico de aborto.

### Tamanho do Estudo

Para a realização da amostragem do estudo, foi utilizado o censo de 2010 do IBGE para selecionar setores censitários que correspondem à unidade cadastral por área contínua contido em uma região, de modo que 108.515 domicílios foram escolhidos na região. Esses domicílios foram divididos em cem, proporcionando a escolha do setor inicial de forma aleatória, e os outros 99 setores subsequentes foram selecionados com base em um pulo sistemático. Após isso, o cálculo chegou a um valor de 1.086 mulheres a serem selecionadas por domicílio. Salienta-se que, no processo de seleção, se houvesse mais de uma mulher elegível na residência, uma lista numérica era sorteada para a seleção aleatória de uma delas. Todo esse processo foi realizado no *software* estatístico R, levando em consideração a classificação do nível socioeconômico. Para o presente estudo, a amostra foi composta por mães de filhos de até 19 anos residentes no município de Vitória, Espírito Santo, totalizando 418 mulheres.

### Métodos Estatísticos

Para a análise descritiva, utilizaram-se frequência absoluta, relativa, média, desvio padrão e Intervalo de Confiança de 95% (IC95%). Na análise bivariada, aplicaram-se o teste qui-quadrado de Pearson (χ^2^) e o teste exato de Fisher, a fim de examinar a relação entre as variáveis. Para testar a correlação entre os itens que compõem as táticas de conflitos, coeficientes Phi (φ) foram calculados devido à natureza das variáveis. A análise de dados foi conduzida no *software* Stata, versão 17.0, e a matriz de correlação foi realizada no *software* R, versão 4.3.1, com ajuste do valor de p <0,05.

A pesquisa foi aprovada pelo Comitê de Ética em Pesquisa da Universidade Federal do Espírito Santo, sob o Parecer nº 4.668206.

## RESULTADOS

As mulheres participantes da presente pesquisa apresentaram uma média de idade de 41 anos e uma escolaridade média de 12 anos de estudo completos. A renda familiar média foi de R$ 4.447,34, com uma mediana de R$ 2.400 reais, indicando uma distribuição assimétrica da variável. Em relação à maternidade, a média de gestações foi de 2,45, e o número médio de filhos foi de 2,16. A média de número de pessoas residentes no domicílio foi de 3,63. Quanto ao histórico reprodutivo, a média de abortos relatados foi de 1,32 (Tabela 1).

**Tabela 1:** Caracterização da amostra de mães participantes do estudo em Vitória, ES, Brasil, 2022 (N = 418).

	Média	Desvio padrão	Mediana	Intervalo
Idade	41,03	± 11,81	40,0	33–47
Escolaridade	12,03	± 3,89	11,0	10–15
Renda familiar (R$)	4447,34	± 5029,67	2400	1250–6000
Número de gestações	2,45	± 1,68	2,0	1–3
Número de filhos	2,16	± 1,60	2,0	1–3
Número de pessoas que vivem na casa	3,63	± 1,36	3,05	3–4
Aborto	1,32	± 0,68	1,0	1–1

Em relação ao desfecho de DNV, a maioria (88,8%) das mães explicou aos filhos o porquê suas ações estavam erradas (IC95%: 85,7–91,8); 76,8% (IC95%: 72,7–80,1) colocaram os filhos de castigo, como no quarto ou outro local; 60,8% (IC95%: 56,1–65,4) deram outra atividade para substituir a ação errada; e 68,9% (IC95%: 64,5–73,3) tiraram benefícios ou restringiram saídas. No que tange à agressão psicológica, 81,8% das mães recorreram à prática de gritar ou berrar (IC95%: 78,1–85,5). Além disso, 14,8% xingaram ou rogaram pragas contra os filhos (IC95%: 11,4–18,2); 9,8% (IC95%: 6,9–12,6) ameaçaram expulsar a criança de casa; 61,7% (IC95%: 57,1–66,4) ameaçaram dar um tapa, mas não o fizeram; e 25,4% (IC95%: 21,2–29,5) chamaram os filhos de burro, estúpido ou similar (Tabela 2).

**Tabela 2:** Táticas de resolução de conflitos em relação à disciplina não violenta e agressão psicológica utilizadas por mães residentes em Vitória, ES, Brasil, 2022 (N = 418).

Disciplina não violenta	n	%	IC95%
Você explicou o porquê o que ele/a estava fazendo estava errado?	371	88,8	85,7–91,8
Você o/a colocou de castigo do tipo: mandou-o ficar em seu quarto ou em qualquer outro lugar?	321	76,8	72,7–80,1
Você deu a ele/a outra coisa para fazer em vez daquilo que ele/a estava fazendo de errado?	254	60,8	56,1–65,4
Você tirou as regalias dele/a ou deixou-o/a sem sair de casa?	288	68,9	64,5–73,3
**Agressão psicológica**	**n**	**%**	**IC95%**
Você falou alto, berrou ou gritou com seu filho?	342	81,8	78,1–85,5
Você xingou ou praguejou, quer dizer, rogou praga, contra ele/a?	62	14,8	11,4–18,2
Você disse alguma vez que iria expulsá-lo/a de casa ou enxotá-lo/a para fora de casa?	41	9,8	6,9–12,6
Você ameaçou dar um tapa nele/a, mas não deu?	258	61,7	57,1–66,4

Legenda: IC95% – Intervalo de Confiança de 95%; n – número; % – porcentagem.

Sobre a punição corporal, 35,9% (IC95%: 31,3–40,5) sacudiram a criança; 56,2% (IC95%: 51,5–61,0) bateram com objetos como cinto ou chinelo; 70,8% (IC95%: 66,5–75,2) deram palmadas no bumbum; 49,5% (IC95%: 44,7–54,3) deram tapas nas mãos, braços ou pernas; e 30,9% deram beliscões (IC95%: 26,4–35,3). Quanto aos maus-tratos físicos, 6,7% (IC95%: 4,3–9,1) bateram com a mão fechada ou chutaram a criança; 23,4% (IC95%: 19,4–27,5) usaram objetos para bater em outras partes do corpo além do bumbum; 2,2% (IC95%: 0,8–3,5) jogaram a criança no chão ou a agarraram pelo pescoço e sacudiram; e cerca de 3% relataram bater na criança de forma contínua e intensa (IC95%: 1,3–4,5) (Tabela 3).

**Tabela 3:** Táticas de resolução de conflitos relacionadas à violência física utilizadas por mães residentes em Vitória, ES, Brasil, 2022 (N = 418).

	Agressão física	n	%	IC95%
Punição corporal	Você o sacudiu?	150	35,9	31,3–40,5
	Você bateu no bumbum dele/a com alguma coisa, como um cinto, chinelo, escova de cabelo, vara ou outro objeto duro?	235	56,2	51,5–61,0
	Você deu uma palmada no bumbum?	296	70,8	66,5–75,2
	Você deu um tapa na mão, no braço ou na perna?	207	49,5	44,7–54,3
	Você o/a deu um beliscão?	129	30,9	26,4–35,3
Maus-tratos físicos	Você bateu com a mão fechada ou deu um chute com força nele/a?	28	6,7	4,3–9,1
	Você bateu em alguma parte do corpo dele/a diferente do bumbum com alguma coisa como um cinto, chinelo, escova de cabelo, vara ou outro objeto duro?	98	23,4	19,4–27,5
	Você jogou no chão?	9	2,2	0,8–3,5
	Você o/a agarrou pelo pescoço e o/a sacudiu?	9	2,2	0,8–3,5
	Você bateu muito, ou seja, bateu nele/a sem parar, o máximo que você conseguiu?	12	2,9	1,3–4,5

Legenda: IC95% – Intervalo de Confiança de 95%; n – número; % – porcentagem.

A Figura 1 apresenta a correlação entre as táticas de conflitos. A análise demonstrou que algumas variáveis apresentam correlação positiva, como “xingou” com “tapa no rosto” (0,40), “bateu no bumbum” com “palmada” (0,58), “bateu no bumbum” com “bateu em outras partes corpo diferente do bumbum” (0,44), “bateu muito” com “jogou no chão” (0,47) e “bateu em outras partes do corpo diferente do bumbum” com “tapa na mão” (0,40).

**Figura 1:** Matriz de correlação entre as táticas de conflitos das mães residentes no município de Vitória, ES, Brasil, 2022 (N = 418).

## DISCUSSÃO

Os achados do presente estudo mostram que a DNV é a prática majoritariamente adotada pelas mães do estudo. O resultado da análise corrobora os de estudo semelhante na região Sul do Brasil com pais de crianças de 4 a 6 anos, onde a prática de DNV por parte das mães também foi a mais prevalente, sendo utilizada por 60% das mães^(22)^.

Outros estudos, como o que analisou as táticas de resolução de conflitos em locais específicos de diversos países, constataram uma taxa de resposta positiva à DNV de até 98%. As Filipinas lideram a prática, com 97% de aceitação, seguidas pelo Chile, com 96%, e posteriormente pelo Brasil, com 96%, com dados da cidade de São Paulo. No entanto, apesar de prevalecerem em todos os países analisados, as práticas positivas vêm acompanhadas de táticas violentas de resolução de conflitos, o que evidencia o tratamento severo das crianças como um problema epidêmico em todas as comunidades analisadas^(23)^.

Ainda, no presente estudo, verifica-se que a maioria das participantes (81,8%) recorreu a pelo menos uma prática de agressão psicológica. Em relação ao abuso psicológico, os resultados corroboram pesquisa nos serviços de emergência da capital do estado do Rio de Janeiro, onde a prevalência de agressão psicológica foi de 94,8%. Os autores chamam a atenção ao fato de que, apesar de haver uma alta prevalência de agressão psicológica, a notificação desse tipo de violência parece quase não ocorrer no estado do Rio de Janeiro^(24)^.

Os resultados identificam uma alta prevalência de violência física, sendo que aproximadamente 71% afirmaram a prática de menos um ato de punição corporal. Estudo semelhante realizado na população portuguesa apresenta uma taxa de 87,7% de resposta positiva para ao menos uma prática de punição corporal, sendo inferior apenas à prática de agressão psicológica na população de sete anos^(25)^. Esses dados mostram que apesar de ser criminalizado no país desde 2007, os castigos corporais estão relacionados, segundo os pais, ao ato de educar^(26)^.

Estudos indicam que a proibição legal por si só tem eficácia limitada, e deve ser acompanhada de ações educativas, assim como da promoção da parentalidade positiva para mudanças efetivas^(27)^. Além disso, outros estudos já puderam comprovar que a punição corporal é um método disciplinar ineficiente que tende a uma obediência imediata, mas não produz autorregulação emocional nem consciência de seus atos^(28)^. Tal fato reforça o fato de que a punição corporal, além de não ser um método coeso de educação, representa uma violação dos direitos da criança e do adolescente, apresentando-se como um problema de saúde pública com consequências duradouras e severas, que demanda políticas integradas de prevenção, apoio psicossocial e fortalecimento das competências parentais^(29)^.

O mesmo é constatado por estudo em Diamantina, cidade mineira localizada no Sudeste do Brasil que constata que a violência é frequentemente acompanhada de justificativas que a tornam naturalizada no cotidiano, sendo interpretada como uma prática comum e banalizada na nossa sociedade. A violência é vista como um instrumento legítimo e aceitável de disciplina, o que contribui para sua perpetuação e dificulta o reconhecimento de seus impactos nocivos^(30)^.

Os maus-tratos estiveram presentes em pelo menos 23,4% das respostas das participantes. Estudo com uma população da região Sudeste do Brasil aponta que 50,3% das mães praticavam ao menos um tipo de maus-tratos com seus filhos como tática de resolução de conflitos^(31)^. Outro estudo na China constatou que a taxa de resposta a práticas de maus-tratos foi de 72,5%, o que sugere que a tolerância ao castigo corporal em crianças no oriente é culturalmente maior em comparação à sociedade ocidental^(32)^.

Vale destacar que apesar da alta prevalência de uso da DNV, a sua prática tem sido observada concomitantemente a outras práticas de táticas de conflitos, como sinalizado pelos autores da CTSPC durante sua validação, reforçando que os pais tendem a usar mais de uma tática para controlar o comportamento da criança. Constatam ainda que a punição corporal é uma prática popular e aceita socialmente^(8)^. Essa constatação é verificada na presente pesquisa, que aponta uma correlação positiva entre as práticas de agressão física e psicológica.

A maioria das famílias combina as práticas de maus-tratos físicos com algum tipo de abuso físico e emocional. Qualquer arranjo de práticas que envolva a prática de maus-tratos merece atenção, independentemente da incidência. Isso porque os maus-tratos infantis têm o maior impacto sobre as crianças, afetando seu prazer de viver e influenciando seu futuro e sua perspectiva^(33,34)^. O profissional de saúde deve estar atento aos indicadores de maus-tratos e suas peculiaridades, para que sejam traçadas estratégias eficientes voltadas à população em questão, com estratégias e políticas eficazes ao combate desse tipo de violência.

O uso de qualquer prática agressiva coercitiva está relacionado a diversos problemas de saúde no decorrer da vida, incluindo problemas de saúde mental, piores relacionamentos entre pais e filhos, uso de substâncias, desenvolvimento socioemocional prejudicado, resultados acadêmicos negativos, problemas de comportamento externalizantes aumentados, entre outros^(35)^.

Por se tratar de estudo transversal, existe a possibilidade de haver viés de memória, uma vez que os dados foram obtidos por meio de autorrelato, dependendo da recordação dos participantes sobre eventos passados, o que pode subestimar os achados. O estudo também está sujeito ao viés de informação, devido ao fornecimento de respostas socialmente desejáveis. Ainda, vale mencionar que se tratam de dados referentes a uma capital do Brasil, e o estudo não é capaz de dimensionar esses dados a nível nacional. Todavia, mesmo diante dessas limitações, os achados são de grande relevância e revelam a magnitude das táticas de conflitos entre mães e filhos e a importância de outros estudos acerca da temática.

## CONCLUSÃO

Embora os resultados revelem a DNV como amplamente adotada, há uma presença significativa de agressão psicológica e física na tática de resolução de conflitos entre mães e filhos, com destaque para punições corporais e práticas de maus-tratos. Esse achado é preocupante, pois enfraquece os vínculos afetivos e pode ter efeitos duradouros na saúde mental e no comportamento da criança, bem como impactar a forma como as crianças lidam com as situações de conflitos por nunca saberem o que esperar dos responsáveis como medida de resolução da problemática. Além disso, a vivência da violência pode prejudicar a capacidade de estabelecer relações saudáveis e seguras, perpetuando ciclos de sofrimento e exclusão social.

Por fim, o presente estudo não só aponta a necessidade de outras pesquisas, como também evidencia a importância de campanhas de sensibilização pública voltadas à divulgação da legislação que proíbe o castigo corporal. Essas campanhas, quando associadas a programas educativos que orientam os pais sobre práticas disciplinares não violentas e respeitosas, configuram-se como elementos promotores de transformações nas crenças e comportamentos dos pais frente ao uso de estratégias disciplinares coercitivas.

## Data Availability

Todo o conjunto de dados que dá suporte aos resultados deste estudo está disponível mediante solicitação ao autor correspondente.

# Translation: Tácticas de resolución de conflictos entre madres e hijos en la capital del sudeste: prevalencias y correlaciones

**DOI:** 10.1590/1980-220X-REEUSP-2025-0351es

**Authors:** Laura Fontes Silva, Franciéle Marabotti Costa Leite

**Affiliations:** 1 Universidade Federal do Espírito Santo, Programa de Pós-Graduação em Saúde Coletiva, Vitória, ES, Brasil.

**Keywords:** Conflicto Familiar, Relaciones Familiares, Abuso Sexual Infantil

## Abstract

**Objetivo:**

Identificar la prevalencia de tácticas de resolución de conflictos entre madres e hijos y sus correlaciones.

**Método:**

Estudio transversal con 418 mujeres de Vitória, Espírito Santo. El resultado fue la táctica de resolución de conflictos utilizada por madres con hijos menores de 19 años, medida mediante la Parent-Child Conflict Tactics Scales, categorizada en disciplina no violenta, agresión psicológica, castigo corporal y maltrato. Los análisis se realizaron con STATA y R, y se presentaron como frecuencia absoluta, frecuencia relativa, media, desviación estándar, intervalo de confianza del 95%, prueba de chi-cuadrado de Pearson (χ^2^) o prueba exacta de Fisher, y prueba de correlación.

**Resultados:**

Se observó una mayor prevalencia de disciplina no violenta (P: 88,8%; IC95%: 85,7–91,8). Aproximadamente el 82% (IC95%: 78,1–85,5) utilizó agresión psicológica, el 35,9% utilizó castigo corporal (IC95%: 31,3–40,5) y el 23,4% (IC95%: 19,4–27,5) utilizó algún método disciplinario que constituye maltrato.

**Conclusión:**

Si bien la disciplina no violenta es ampliamente adoptada por las madres, existe una presencia significativa de agresión psicológica y física, así como de prácticas de maltrato.

## INTRODUCCIÓN

Aunque la parentalidad ha experimentado cambios en la actualidad, la responsabilidad principal de la crianza de los hijos sigue recayendo predominantemente en las madres^(1)^. La maternidad tal y como la vemos hoy en día es el resultado de cambios en el contexto familiar que atribuyen a la mujer el papel no solo de nutrir, sino también de cuidar y orientar a sus hijos, siendo, en la mayoría de los casos, la principal cuidadora^(2)^. Se trata, por lo tanto, de un concepto complejo que no se limita a una dimensión específica, sino que se caracteriza como una adaptación multidimensional en la vida de la mujer que implica la redefinición de la imagen corporal y la identidad, la formación del apego, el bienestar, el cambio de prioridades en las relaciones y la confianza en el desempeño del papel de madre, lo que permite a la mujer integrarse y equilibrarse tanto como individuo como madre^(3)^.

La alta demanda, el comportamiento del niño y el período de adaptación al nuevo papel hacen que el inicio de la jornada parental sea un reto para los padres^(4)^. La comprensión de que la forma en que los principales cuidadores manejan sus propios problemas ante el cuidado de los hijos influye en la forma en que los niños desarrollan sus habilidades emocionales y sociales^(5)^, lo que culmina en nuevas producciones en materia de salud que orientan a los padres hacia prácticas parentales más conscientes y basadas en el respeto mutuo^(6)^.

Es evidente que, en contextos difíciles como los de conflicto, la habilidad emocional se convierte en una cuestión central^(7)^. Los conflictos son inherentes a las relaciones humanas^(8)^, y el entorno familiar suele ser un espacio propicio para el surgimiento de desacuerdos, debido a las inevitables divergencias y tensiones cotidianas entre sus miembros^(9)^. Los conflictos recurrentes entre padres e hijos se caracterizan por discusiones frecuentes e irresolubles entre padres e hijos sobre los mismos temas^(10)^.

Los motivos por los que las madres tienen conflictos relacionados con sus hijos van cambiando a lo largo del desarrollo del niño, como se constató en un estudio con jóvenes de 15 a 24 años que identificó que los principales conflictos parentales son entre madres e hijos, siendo la principal motivación las tareas domésticas, seguidas por el dinero y el uso de Internet. En la investigación también se presentan otras motivaciones, como la amistad/noviazgo, las drogas y los estudios^(11)^. Es un hecho que los conflictos frecuentes pueden generar estrés tóxico, perjudicando el desarrollo emocional y cognitivo, y contribuyendo a trastornos mentales en ambos implicados^(12)^. Estos conflictos suelen estar asociados a ciclos intergeneracionales de violencia y negligencia^(13)^.

En este contexto, el Estatuto del Niño y del Adolescente y la Ley n.º 13.010/2014 son fundamentales, ya que garantizan el derecho a la educación y al cuidado sin el uso de castigos físicos o tratos crueles y degradantes a todos los niños y adolescentes, reconociendo la violencia doméstica como una violación de los derechos, y no como una práctica educativa legítima^(14,15)^. Cabe mencionar que, en 2014, se sancionó en Brasil la Ley de la Palmada, por la que se tipificó como delito cualquier castigo físico con intencionalidad punitiva o disciplinaria y, del mismo modo, las conductas crueles que puedan degradar, intimidar o humillar a las víctimas^(15)^.

Sin embargo, a pesar de los esfuerzos del Estado por implementar medidas que protejan a los niños y adolescentes en situación de violencia y negligencia, el fenómeno no ha hecho más que aumentar con el paso de los años.

Según el Anuario Brasileño de Seguridad Pública, en el primer semestre de 2021, Brasil ya había registrado más de 50 000 denuncias de violencia contra niños y adolescentes en el territorio nacional a través del “Disque 100”, canal de denuncia de violaciones de los derechos humanos en el país. En el 81 % de las denuncias, los actos de violencia tuvieron lugar en el hogar de las víctimas, y los principales agresores fueron las madres, seguidas de los padres^(16)^. En Espírito Santo, entre 2011 y 2018, se notificaron 3127 casos de violencia contra menores, siendo los agresores, en su mayoría, personas con vínculo materno y paterno con la víctima^(17)^.

Es importante reflexionar sobre el hecho de que el uso del castigo físico en los niños puede modificarse mediante intervenciones específicas basadas en programas de apoyo parental que enseñan alternativas a la crianza sin violencia^(18)^. La Ley n.º 14.826/2024 ofrece orientación sobre la parentalidad positiva y el derecho al juego como herramientas para prevenir la violencia contra los niños. Las iniciativas basadas en criterios de respeto, comunicación y límites se utilizan en la asistencia social, la educación, la cultura, la salud y la seguridad pública en todo el país (Proyecto de Ley n.º 2.861/2023)^(19)^.

En vista de lo anterior, comprender las tácticas utilizadas por las madres para resolver los conflictos domésticos es fundamental para la salud pública, ya que repercute directamente en el bienestar de los niños y en la dinámica familiar. Conocer estos patrones puede ayudar a prevenir la violencia doméstica y a promover la salud mental, ofreciendo un apoyo adecuado a las madres en situaciones de estrés. En este sentido, el presente estudio busca identificar la prevalencia de las tácticas de resolución de conflictos entre madres e hijos y sus correlaciones.

## MÉTODO

### Diseño del Estudio

Se trata de una investigación transversal, descriptiva y analítica, derivada de un estudio poblacional realizado en el municipio de Vitória, Espírito Santo, Brasil.

### Contexto

El municipio de Vitória, capital del estado de Espírito Santo, según el censo del Instituto Brasileño de Geografía y Estadística (IBGE) de 2022, tiene una superficie de 97,123 km^2^ y una población residente de 322,869 personas; de las cuales el 54 % son mujeres, lo que indica un total aproximado de 174 348 mujeres residentes, distribuidas en 80 barrios con una densidad demográfica de 3324,33 hab./km^2(20)^.

El estudio de base poblacional se llevó a cabo en el año 2022, de enero a mayo, y el estudio piloto en 2021. La recolección de datos se realizó en los domicilios por equipos compuestos por entrevistadoras y supervisores. Se realizaron entrevistas a las mujeres utilizando un cuestionario estructurado como guía, aplicado cara a cara en el domicilio de la mujer, garantizando la privacidad.

Los datos recopilados se computaron en tabletas y se gestionaron con la ayuda de la herramienta electrónica de captura de datos Research Eletronic Data Capture.

### Participantes

La población objetivo del estudio incluyó a mujeres de 18 años o más que tenían o habían tenido una pareja íntima en los 24 meses anteriores a la entrevista y que tenían hijos de hasta 19 años de edad. La pareja íntima se definió como la pareja actual o anterior, siempre que hubiera habido relación sexual, independientemente de que estuvieran unidos formalmente. Como criterio de exclusión, se consideraron las mujeres que presentaban limitaciones cognitivas derivadas de déficits intelectuales o sensoriales que les impedían responder al instrumento de recolección de datos.

### Variables

El resultado del estudio son los conflictos entre madres e hijos, que se identificaron mediante el uso de las Escalas de Tácticas de Conflicto entre Padres e Hijos (*Parent-Child Conflict Tactics Scales*- CTSPC), que constan originalmente de 22 ítems que investigan las reacciones al conflicto a partir del comportamiento parental de los padres hacia los hijos. La escala está validada para su uso en Brasil. Se evalúan tres dimensiones: disciplina no violenta (DNV) - (cuatro ítems) táctica relativa a métodos de corrección basados en explicaciones, diálogo y retirada de privilegios, sin uso de la fuerza física; agresión psicológica (cinco ítems), constituida por actos verbales y simbólicos que causan miedo, humillación o dolor emocional en el niño, como gritar, amenazar e insultar; violencia física (13 ítems), que comprende el uso de la fuerza corporal, desde castigos leves, como palmadas, hasta agresiones graves, que constituyen maltrato. Esta última se subdividió según su gravedad en castigo corporal (seis ítems), maltrato físico (tres ítems) y maltrato físico grave (cuatro ítems)^(21)^.

Para el presente estudio, la medición de cada dominio se realizó de forma dicotómica (presencia/ausencia de la conducta), con opciones de respuesta “sí” o “no”. Se excluyeron dos preguntas relacionadas con el maltrato físico grave. De este modo, en la presente investigación, la escala contempla 20 ítems preguntados. La exclusión de las dos preguntas relacionadas con el maltrato físico grave se produjo tras la prueba piloto, ya que las entrevistadoras constataron que las preguntas relacionadas con el uso de armas de fuego y el uso de líquidos calientes para herir a sus hijos generaban incomodidad y desconfianza en la participación en el estudio. Por lo tanto, se excluyeron del cuestionario.

En relación con las variables independientes, se evaluaron las siguientes características: edad (18–29; 30–39; 40–49; 50–59; 60 o más); escolaridad en años de estudio (0–8; 9–11; 12 o más); ingresos familiares (1er tercil: más pobres; 2.º tercil y 3.º tercil: más rico); número de residentes en el domicilio (viviendo solo; dos; tres; cuatro; cinco o más personas); número de embarazos (uno; dos; tres; cuatro o más), número de hijos (uno; dos; tres; cuatro o más); historial de abortos.

### Tamaño del Estudio

Para realizar el muestreo del estudio, se utilizó el censo de 2010 del IBGE para seleccionar los sectores censales que corresponden a la unidad catastral por área continua contenida en una región, de modo que se eligieron 108 515 hogares en la región. Estos hogares se dividieron en cien, lo que permitió la selección aleatoria del sector inicial, y los otros 99 sectores posteriores se seleccionaron mediante un salto sistemático.

A continuación, se calculó que se seleccionarían 1086 mujeres por hogar. Cabe destacar que, en el proceso de selección, si había más de una mujer elegible en la residencia, se sorteaba una lista numérica para la selección aleatoria de una de ellas. Todo este proceso se llevó a cabo en el software estadístico R, teniendo en cuenta la clasificación del nivel socioeconómico. Para el presente estudio, la muestra estuvo compuesta por madres de hijos de hasta 19 años residentes en el municipio de Vitória, Espírito Santo, con un total de 418 mujeres.

### Métodos Estadísticos

Para el análisis descriptivo, se utilizaron la frecuencia absoluta, relativa, media, desviación estándar e intervalo de confianza del 95 % (IC95 %). En el análisis bivariado, se aplicaron la prueba de Chi-cuadrado de Pearson (χ^2^) y la prueba exacta de Fisher, con el fin de examinar la relación entre las variables. Para probar la correlación entre los ítems que componen las tácticas de conflicto, se calcularon los coeficientes Phi (φ) debido a la naturaleza de las variables. El análisis de datos se realizó con el software Stata, versión 17.0, y la matriz de correlación se realizó con el software R, versión 4.3.1, con un ajuste del valor de p <0,05.

La investigación fue aprobada por el Comité de Ética en Investigación de la Universidad Federal de Espírito Santo, bajo el dictamen n.º 4.668206.

## RESULTADOS

Las mujeres que participaron en la presente investigación tenían una edad media de 41 años y una escolaridad media de 12 años completos de estudios. La renta familiar media fue de 4447,34 reales, con una mediana de 2400 reales, lo que indica una distribución asimétrica de la variable. En relación con la maternidad, el promedio de embarazos fue de 2,45 y el promedio de hijos fue de 2,16. El promedio de personas residentes en el domicilio fue de 3,63. En cuanto al historial reproductivo, el promedio de abortos reportados fue de 1,32 (Tabla 1).

**Tabla 1:** Caracterización de la muestra de madres participantes en el estudio en Vitória, ES, Brasil, 2022 (N = 418).

	Media	Desvío estándar	Mediana	Intervalo
Edad	41,03	± 11,81	40,0	33–47
Escolaridad	12,03	± 3,89	11,0	10–15
Ingreso familiar (Reales)	4447,34	± 5029,67	2400	1250–6000
Número de gestaciones	2,45	± 1,68	2,0	1–3
Número de hijos	2,16	± 1,60	2,0	1–3
Número de personas que viven en la casa	3,63	± 1,36	3,05	3–4
Aborto	1,32	± 0,68	1,0	1–1

En cuanto al resultado del DNV, la mayoría (88.8 %) de las madres explicaron a sus hijos por qué sus acciones eran incorrectas (IC95 %: 85.7–91.8); el 76.8 % (IC95 %: 72.7–80.1) castigaron a sus hijos, por ejemplo, enviándolos a su habitación u otro lugar; el 60.8 % (IC 95 %: 56.1–65.4) les dio otra actividad para sustituir la acción incorrecta; y el 68.9 % (IC 95 %: 64.5–73.3) les quitó privilegios o restringió las salidas. En cuanto a la agresión psicológica, el 81.8 % de las madres recurrieron a gritar o vociferar (IC95 %: 78.1–85.5). Además, el 14.8 % insultó o maldijo a sus hijos (IC95 %: 11.4–18.2); el 9.8 % (IC95 %: 6.9–12.6) amenazó con echar al niño de casa; el 61.7 % (IC95 %: 57.1–66.4) amenazó con dar una bofetada, pero no lo hizo; y el 25.4 % (IC95 %: 21.2–29.5) llamó a sus hijos tontos, estúpidos o similares (Tabla 2).

**Tabla 2:** Tácticas de resolución de conflictos en relación con la disciplina no violenta y la agresión psicológica utilizadas por madres residentes en Vitória, ES, Brasil, 2022 (N = 418).

Disciplina no violenta	n	%	IC95%
¿Le explicaste por qué lo que estaba haciendo estaba mal?	371	88,8	85,7–91,8
¿Le impusiste un castigo del tipo: le ordenaste quedarse en su habitación o en cualquier otro lugar?	321	76,8	72,7–80,1
¿Le diste otra cosa que hacer en lugar de lo que estaba haciendo mal?	254	60,8	56,1–65,4
¿Le quitaste privilegios o le impediste salir de casa?	288	68,9	64,5–73,3
**Agresión psicológica**	**n**	**%**	**IC95%**
¿Le ha hablado en voz alta, le ha gritado o le ha chillado a su hijo?	342	81,8	78,1–85,5
¿Le ha insultado o maldecido, es decir, le ha echado maldiciones?	62	14,8	11,4–18,2
¿Le ha dicho alguna vez que le echaría de casa o le echaría a la calle?	41	9,8	6,9–12,6
¿Le ha amenazado con darle una bofetada, pero no lo ha hecho?	258	61,7	57,1–66,4

Siglas: IC95% – Intervalo de Confianza de 95%; n – número; % – porcentaje.

En cuanto al castigo físico, el 35.9 % (IC 95 %: 31.3–40.5) sacudió al niño; el 56.2 % (IC 95 %: 51.5–61.0) golpeó con objetos como cinturones o sandalias; el 70.8 % (IC 95 %: 66.5–75. 2) le dieron palmadas en el trasero; el 49.5 % (IC95 %: 44.7–54.3) le dio palmadas en las manos, los brazos o las piernas; y el 30.9 % le pellizcó (IC95 %: 26.4–35.3). En cuanto al maltrato físico, el 6.7% (IC95%: 4.3–9.1) golpeó con la mano cerrada o pateó al niño; el 23.4% (IC95%: 19.4–27.5) utilizó objetos para golpear otras partes del cuerpo además del trasero; el 2,2 % (IC95 %: 0,8–3,5) tiró al niño al suelo o lo agarró por el cuello y lo sacudió; y alrededor del 3 % informó haber golpeado al niño de forma continua e intensa (IC95 %: 1,3–4,5) (Tabla 3).

**Tabla 3:** Tácticas de resolución de conflictos relacionadas con la violencia física utilizadas por madres residentes en Vitória, ES, Brasil, 2022 (N = 418).

	Agresión física	n	%	IC95%
Castigo corporal	¿Lo zamarreaste?	150	35,9	31,3–40,5
	¿Le pegaste en el trasero con algo, como un cinturón, una sandalia, un cepillo, un palo u otro objeto duro?	235	56,2	51,5–61,0
	¿Le diste una palmada en el trasero?	296	70,8	66,5–75,2
	¿Le diste una palmada en la mano, el brazo o la pierna?	207	49,5	44,7–54,3
	¿Le diste un pellizco?	129	30,9	26,4–35,3
Malos-tratos físicos	¿Le pegaste con la mano cerrada o le diste una patada fuerte?	28	6,7	4,3–9,1
	¿Le pegaste en alguna parte del cuerpo que no fuera el trasero con algo como un cinturón, una sandalia, un cepillo para el cabello, un palo u otro objeto duro?	98	23,4	19,4–27,5
	¿Lo tiraste al suelo?	9	2,2	0,8–3,5
	¿Lo agarraste por el cuello y lo sacudiste?	9	2,2	0,8–3,5
	¿Le pegaste mucho, es decir, le pegaste sin parar, con toda la fuerza que pudiste?	12	2,9	1,3–4,5

Siglas: IC95% – Intervalo de Confianza de 95%; n – número; % – porcentaje.

La Figura 1 muestra la correlación entre las tácticas de conflicto. El análisis demostró que algunas variables presentan una correlación positiva, como “insultó” con “dio bofetada” (0,40), “golpeó en el trasero” con “palmada” (0,58), “golpeó en el trasero” con “golpeó en otras partes del cuerpo distintas del trasero” (0,44), “golpeó mucho” con “tiró al suelo” (0,47) y “golpeó en otras partes del cuerpo distintas del trasero” con “di bofetada” (0,40).

**Figura 1:** Matriz de correlación entre las tácticas de conflicto de las madres residentes en el municipio de Vitória, ES, Brasil, 2022 (N = 418).

## DISCUSIÓN

Los resultados del presente estudio muestran que la DNV es la práctica más adoptada por las madres del estudio. El resultado del análisis corrobora los de un estudio similar realizado en la región sur de Brasil con padres de niños de 4 a 6 años, donde la práctica de la DNV por parte de las madres también fue la más prevalente, siendo utilizada por el 60 % de las madres^(22)^.

Otros estudios, como el que analizó las tácticas de resolución de conflictos en lugares específicos de varios países, constataron una tasa de respuesta positiva a la DNV de hasta el 98 %. Filipinas lidera la práctica, con un 97 % de aceptación, seguida de Chile, con un 96 %, y posteriormente de Brasil, con un 96 %, con datos de la ciudad de São Paulo. Sin embargo, a pesar de prevalecer en todos los países analizados, las prácticas positivas van acompañadas de tácticas violentas de resolución de conflictos, lo que pone de manifiesto el trato severo a los niños como un problema epidémico en todas las comunidades analizadas^(23)^.

Además, en el presente estudio se observa que la mayoría de las participantes (81,8 %) recurrió al menos a una práctica de agresión psicológica. En relación con el abuso psicológico, los resultados corroboran una investigación realizada en los servicios de emergencia de la capital del estado de Río de Janeiro, donde la prevalencia de la agresión psicológica fue del 94.8 %. Los autores llaman la atención sobre el hecho de que, a pesar de la alta prevalencia de la agresión psicológica, la notificación de este tipo de violencia parece ser casi inexistente en el estado de Río de Janeiro^(24)^.

Los resultados identifican una alta prevalencia de violencia física, ya que aproximadamente el 71 % afirmó haber practicado al menos un acto de castigo corporal. Un estudio similar realizado en la población portuguesa presenta una tasa del 87,7 % de respuestas positivas a al menos una práctica de castigo corporal, solo inferior a la práctica de agresión psicológica en la población de siete años^(25)^. Estos datos muestran que, a pesar de estar penalizado en el país desde 2007, los castigos corporales están relacionados, según los padres, con el acto de educar^(26)^.

Los estudios indican que la prohibición legal por sí sola tiene una eficacia limitada y debe ir acompañada de acciones educativas, así como de la promoción de la parentalidad positiva para lograr cambios efectivos^(27)^. Además, otros estudios ya han podido demostrar que el castigo corporal es un método disciplinario ineficaz que tiende a la obediencia inmediata, pero no produce autorregulación emocional ni conciencia de sus actos^(28)^. Este hecho refuerza la idea de que el castigo físico, además de no ser un método educativo coherente, representa una violación de los derechos del niño y del adolescente, lo que lo convierte en un problema de salud pública con consecuencias duraderas y graves, que exige políticas integradas de prevención, apoyo psicosocial y fortalecimiento de las competencias parentales^(29)^.

Lo mismo se constata en un estudio realizado en Diamantina, una ciudad de Minas Gerais, situada en el sureste de Brasil, que revela que la violencia suele ir acompañada de justificaciones que la naturalizan en la vida cotidiana, interpretándola como una práctica común y banalizada en nuestra sociedad. La violencia se considera un instrumento legítimo y aceptable de disciplina, lo que contribuye a su perpetuación y dificulta el reconocimiento de sus efectos nocivos^(30)^.

Los malos tratos estuvieron presentes en al menos el 23,4 % de las respuestas de las participantes. Un estudio realizado con una población del sureste de Brasil señala que el 50,3 % de las madres practicaban al menos un tipo de maltrato con sus hijos como táctica de resolución de conflictos^(31)^. Otro estudio realizado en China constató que la tasa de respuesta a las prácticas de maltrato fue del 72,5 %, lo que sugiere que la tolerancia al castigo corporal en los niños en Oriente es culturalmente mayor en comparación con la sociedad occidental^(32)^.

Cabe destacar que, a pesar de la alta prevalencia del uso de la DNV, su práctica se ha observado concomitantemente con otras prácticas de tácticas de conflicto, como señalaron los autores de la CTSPC durante su validación, lo que refuerza que los padres tienden a utilizar más de una táctica para controlar el comportamiento del niño. También constatan que el castigo corporal es una práctica popular y socialmente aceptada^(8)^. Esta constatación se verifica en la presente investigación, que señala una correlación positiva entre las prácticas de agresión física y psicológica.

La mayoría de las familias combinan las prácticas de maltrato físico con algún tipo de abuso físico y emocional. Cualquier combinación de prácticas que implique el maltrato merece atención, independientemente de su incidencia. Esto se debe a que el maltrato infantil tiene un mayor impacto en los niños, afectando su disfrute de la vida e influyendo en su futuro y sus perspectivas^(33,34)^. Los profesionales de la salud deben estar atentos a los indicadores de maltrato y sus peculiaridades, a fin de diseñar estrategias eficaces dirigidas a la población en cuestión, con estrategias y políticas eficaces para combatir este tipo de violencia.

El uso de cualquier práctica coercitiva agresiva está relacionado con diversos problemas de salud a lo largo de la vida, incluyendo problemas de salud mental, peores relaciones entre padres e hijos, consumo de sustancias, desarrollo socioemocional perjudicado, resultados académicos negativos, aumento de los problemas de conducta externalizantes, entre otros^(35)^.

Al tratarse de un estudio transversal, existe la posibilidad de que haya un sesgo de memoria, ya que los datos se obtuvieron mediante autoinformes, dependiendo de los recuerdos de los participantes sobre eventos pasados, lo que puede subestimar los hallazgos. El estudio también está sujeto a un sesgo de información, debido al suministro de respuestas socialmente deseables. Además, cabe mencionar que se trata de datos referidos a una capital de Brasil, y el estudio no es capaz de extrapolar estos datos a nivel nacional. Sin embargo, incluso ante estas limitaciones, los hallazgos son de gran relevancia y revelan la magnitud de las tácticas de conflicto entre madres e hijos y la importancia de otros estudios sobre el tema.

## CONCLUSIÓN

Aunque los resultados revelan que la DNV es ampliamente adoptada, existe una presencia significativa de agresión psicológica y física en la táctica de resolución de conflictos entre madres e hijos, con especial énfasis en los castigos corporales y las prácticas de maltrato.

Este hallazgo es preocupante, ya que debilita los vínculos afectivos y puede tener efectos duraderos en la salud mental y el comportamiento del niño, así como impactar en la forma en que los niños manejan las situaciones de conflicto al no saber nunca qué esperar de los responsables como medida de resolución del problema. Además, la experiencia de la violencia puede perjudicar la capacidad de establecer relaciones saludables y seguras, perpetuando ciclos de sufrimiento y exclusión social.

Por último, el presente estudio no solo señala la necesidad de realizar otras investigaciones, sino que también pone de manifiesto la importancia de las campañas de sensibilización pública destinadas a difundir la legislación que prohíbe el castigo corporal. Estas campañas, cuando se asocian a programas educativos que orientan a los padres sobre prácticas disciplinarias no violentas y respetuosas, se configuran como elementos promotores de transformaciones en las creencias y comportamientos de los padres frente al uso de estrategias disciplinarias coercitivas.

## Data Availability

El conjunto de datos que respaldan las conclusiones de este estudio está disponible previa solicitud al autor correspondiente.
